# The PD-1/PD-L1 Inhibitory Pathway is Altered in Primary Glomerulonephritides

**DOI:** 10.1007/s00005-017-0485-3

**Published:** 2017-08-02

**Authors:** Ewelina Grywalska, Iwona Smarz-Widelska, Ewelina Krasowska-Zajac, Izabela Korona-Glowniak, Karolina Zaluska-Patel, Michal Mielnik, Martyna Podgajna, Anna Malm, Jacek Rolinski, Wojciech Zaluska

**Affiliations:** 1 Department of Clinical Immunology and Immunotherapy, Medical University of Lublin, Chodzki 4a, 20-093 Lublin, Poland; 2 Department of Nephrology, Cardinal Stefan Wyszynski Provincial Hospital in Lublin, Lublin, Poland; 3 Department of Pharmaceutical Microbiology, Medical University of Lublin, Lublin, Poland; 4 Department of Didactics and Medical Simulation, Medical University of Lublin, Lublin, Poland; 5 Department of Nephrology, Medical University of Lublin, Lublin, Poland

**Keywords:** Glomerulonephritis, Immunology, IgA nephropathy, Immunosuppression, Membranoproliferative glomerulonephritis, Minimal change disease

## Abstract

The pathogenesis of primary proliferative and non-proliferative glomerulonephritides (PGN and NPGN) is still not fully understood, however, current evidence suggests that most cases of PGN and NPGN are the results of immunologic response to different etiologic agents that activates various biological processes leading to glomerular inflammation and injury. Programmed cell death protein 1 (PD-1) is the major inhibitory receptor regulating T cell exhaustion. The aim of this study was to evaluate the frequencies of PD-1-positive and PD-ligand 1 (PD-L1)-positive T and B lymphocytes in patients with NPGN and PGN in relation to clinical parameters for the first time. The study included peripheral blood (PB) samples from 20 newly diagnosed PGN and NPGN patients. The control group comprised of 20 healthy age- and sex-matched subjects. The viable PB lymphocytes underwent labelling with fluorochrome-conjugated monoclonal antibodies anti-PD-1 and anti-PD-L1, and were analyzed using a flow cytometer. The frequencies of CD4^+^/PD1^+^ T lymphocytes, CD8^+^/PD1^+^ T lymphocytes, and CD19^+^/PD-1^+^ B lymphocytes in the PGN group exceeded values obtained both in the NPGN group, and the control group. Alteration of PD-1/PD-L1 pathway may be involved in poorer prognosis, as patients with PGN are characterized by higher frequencies of PD-1-positive and PD-L1-positive T and B lymphocytes than patients with NPGN. Our results suggest that deregulation of PD-1/PD-L1 axis may contribute to the PGN and NPGN pathogenesis. High percentages of lymphocytes with PD-1 and PD-L1 expression may be related to the continuous T-cell activation and development of glomerular inflammation and injury.

## Introduction

The prevalence of chronic kidney disease (CKD) in the United States in 2012 (not including end-stage renal disease: ESRD) was estimated at 13.6% (Saran et al. 2016). According to Centres for Disease Control and Prevention kidney diseases (nephritis, nephrotic syndrome, and nephrosis) are the 9th biggest cause of death in the USA. Primary glomerulonephritides (GN) are among the most frequent causes of ESRD (Ozturk et al. 2014). According to the United States Renal Data System, primary glomerular diseases comprise 12% of the aetiologies of ESRD. Recent Polish data are in line with international findings (Perkowska-Ptasinska et al. 2016). The pathogenesis of proliferative and non-proliferative GN (PGN and NPGN, respectively) is still not fully understood, however, current evidence suggests, that most cases of GN are results of immunologic response to different etiologic agents (Floege 2013; Rodrigues et al. 2014). Humoral response, which is regulated by the T helper 2 (Th2) cell acts by B-cell activation, immunoglobulin deposition, and complement activation in glomeruli. The cellular, Th1-regulated immune response contributes to both the infiltration of circulating mononuclear inflammatory cells (including lymphocytes and macrophages) into glomeruli and to crescent formation (Krebs and Steinmetz 2016).

Programmed cell death protein 1, also known as PD-1 and CD279 is a protein expressed on T cells and pro-B cells in humans. It is a cell surface receptor that belongs to the immunoglobulin superfamily (Odorizzi et al. 2015). PD-1 is known to be the major inhibitory receptor that functions as an immune checkpoint, playing an important role in down regulating the immune system; by preventing T-cell activation it reduces autoimmunity and promotes self-tolerance. However, it has also been proven that T cells with high PD-1 expression lose the ability to eliminate cancer and infectious agents (Chen et al. 2014; Lim et al. 2015; McKay et al. 2015). PD-1 is the major inhibitory receptor regulating T-cell exhaustion, i.e. a state of T-cell dysfunction. While PD-1 is not expressed on naïve T cells, it is unregulated following T-cell receptor (TCR)-mediated activation and readily observed on both activated and exhausted T cells (Chen et al. 2014). Upon ligation of PD-1 by its ligands (PD-L1 and PD-L2), it binds to the TCR complex and leads to inhibition of CD3ζ chain phosphorylation; it results in an overall inhibition of TCR signalling during antigen presentation to naive T cells (Jiang et al. 2016). PD-1/PD-L1/2 pathway exerts critical inhibitory functions in the setting of persistent auto- or exo-antigenic stimulation (Francisco et al. 2010; Jiang et al. 2016). Many studies have suggested that PD-1 and its ligands play an important immunoregulatory function, however, little is known about PD-1/PD-L1 pathway in the pathogenesis and progression of primary GN.

The aim of this preliminary study was to evaluate the frequencies of PD-1-positive and PD-L1-positive T and B lymphocytes in patients with NPGN and PGN in relation to clinical parameters.

## Materials and Methods

### Characteristics of Patients and Healthy Volunteers

The study included peripheral blood (PB) samples from 20 newly diagnosed, previously untreated patients with primary glomerulonephritis (11 men and 9 women). Ten patients were diagnosed with PGN (seven patients with IgA nephropathy, and three patients with membranoproliferative glomerulonephritis), and ten patients were diagnosed with NPGN (four patients with minimal change disease, and six patients with membranous glomerulonephritis). The mean age of patients from the study groups was 42.4 ± 12.2 years [median 42.5 years (21–62 years)] in PGN patients, and 45.7 ± 13.4 years [median 50.0 years (23–63 years)] in NPGN patients. The control group comprised 20 healthy subjects (12 men and 8 women), aged 44.4 ± 12.2 years [median 45.0 years (20–61 years)].

Neither the patients nor the controls used immunomodulating agents or hormonal preparations, showed signs of infection within at least three months prior to the study, underwent blood transfusion, or presented with autoimmune condition or allergy. Moreover, none of the controls had a history of oncological therapy or prior treatment for tuberculosis or other chronic conditions that could be associated with impaired cellular or humoral immunity.

The diagnosis of primary glomerulonephritides was established on the basis of standard diagnostic criteria, with special emphasis on kidney biopsy (Floege and Amann 2016).

This study was approved by the Ethics Committee of the Medical University of Lublin (Decision No. KE-0254/290/2014). Written informed consent was obtained from all patients with respect to the use of their blood for scientific purposes.

### Isolation of PB Cells and the Detection of PD-1-Positive and PD-L1-Positive T and B Lymphocytes

Venous blood samples in an amount of 5 ml were collected from the study patients and controls by venipuncture using sterile, lithium heparin-treated tubes (S-Monovette, SARSTEDT, Aktiengesellschaft & Co., 51588 Nubrecht, Germany).

**Fig. 1:**
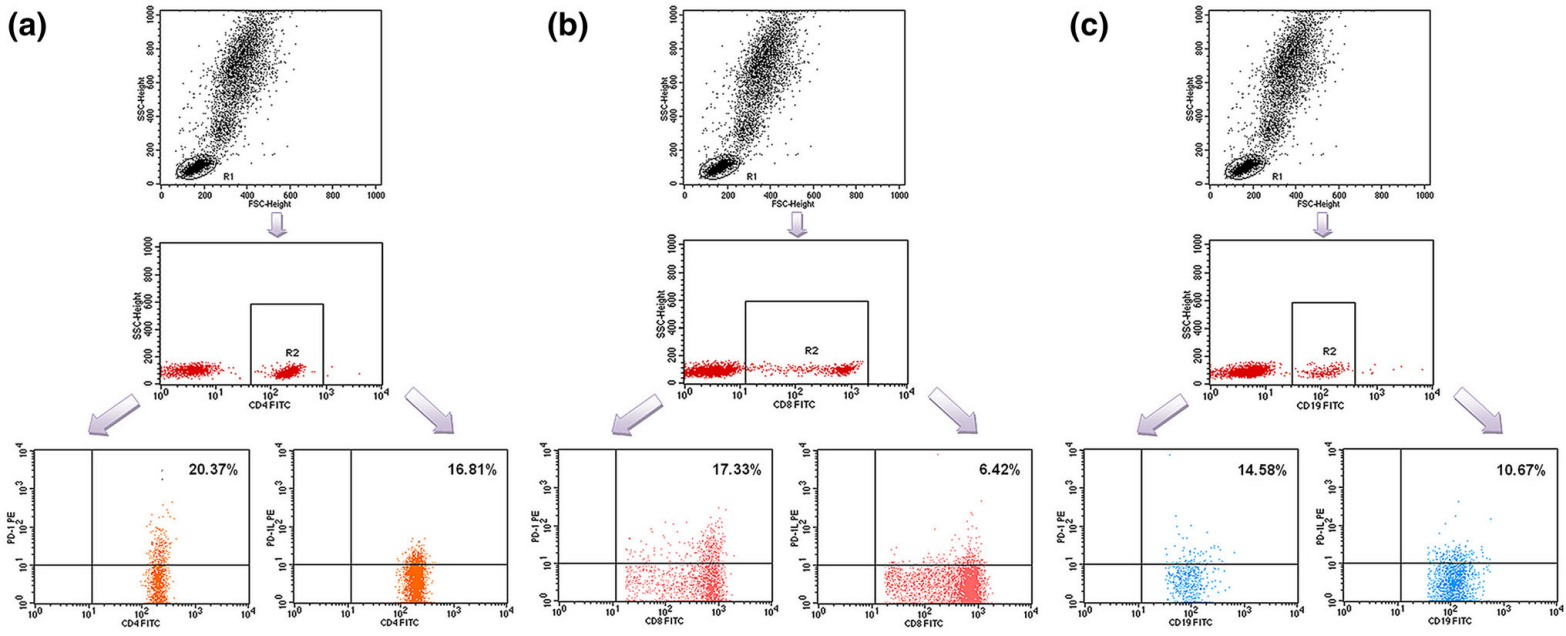
**a** Sample analysis of PD-1^+^/CD4^+^ T lymphocytes and PD-L1^+^/CD4^+^ T lymphocytes in a patient with PGN. **b** Sample analysis of PD-1^+^/CD8^+^ T lymphocytes and PD-L1^+^/CD8^+^ T lymphocytes in a patient with PGN. **c** Sample analysis of PD-1^+^/CD19^+^ B lymphocytes and PD-L1^+^/CD19^+^ B lymphocytes in a patient with PGN

Peripheral blood mononuclear cells were aseptically separated by a standard density gradient centrifugation 1 h after obtaining from the patients and controls (Gradisol L, Aqua Medica, Poland). The percentages of cells expressing surface markers were analyzed. The cells were phenotypically characterized by incubation (20 min in the dark at room temperature) with combination of relevant fluorescein isothiocyanate (FITC)—phycoerythrin (PE)—and CyChrome-labelled monoclonal antibodies (mAbs). Immunofluorescence studies were performed using a combination of the following mAbs: CD45 FITC/CD14 PE, CyChrome Mouse Anti-Human CD3, FITC Mouse Anti-Human CD19, FITC Mouse Anti-Human CD4, FITC Mouse Anti-Human CD8, PE Mouse Anti-Human CD279 (PD-1) and PE Mouse Anti-Human CD274 (PD-L1), purchased from BD Biosciences (USA). Three-colour immunofluorescence analyses were performed using a FACSCalibur flow cytometer (Becton–Dickinson) equipped with 488 nm argon laser. A minimum of 10,000 events was acquired and analyzed using CellQuest Software. The results were presented as percentage of CD45^+^ cells stained with antibody. The percentage of positive cells was calculated by comparing with the control. Background fluorescence was determined using isotype-matched directly conjugated FITC Mouse IgG1 κ Isotype Control and PE Mouse IgG1 κ Isotype Control monoclonal antibodies. The samples were gated on forward scatter vs. side scatter to exclude debris and cell aggregates. Example of cytometric analysis is presented on the Fig. 1.

### Statistical Analysis

Normal distribution of continuous variables was tested using the Shapiro–Wilk test. Statistical characteristics of the continuous variables were presented as medians, minimum and maximum values, as well as arithmetic means and their standard deviations (SD). The Student *t* test was used for independent variables, and the Mann–Whitney *U* test was used for intergroup comparisons. The power and direction of relationships between pairs of continuous variables were determined on the basis of the values of Spearman’s coefficient of rank correlation. The distributions of discrete variables in the studied groups were compared with the Pearson’s Chi-square test or the Fisher’s exact test. Receiver operating characteristic (ROC) curves was generated for significant predictor variables of PGN patients. Areas under the ROC curves (AUCs) were calculated for each parameter and compared. All the calculations were carried out with Statistica 10 (StatSoft^®^, USA) package and Graphpad Prism 5 (Graphpad Software, Inc.). Differences were considered statistically significant with a *p* value <0.05.

## Results

### Basic Characteristics of Patients and Controls

Complete blood count parameters of patients from study groups and controls are presented in Table 1. Table 2 presents a comparison of renal function parameters in PGN patients, NPGN patients and control group. Levels of selected blood proteins and complement components in PGN patients, NPGN patients and healthy individuals are presented in the Table 3.

**Table 1:** Complete blood count parameters of patients from study groups and controls

Parameters	PGN		NPGN		Control group		Group of patients					
							PGN vs. NPGN		PGN vs. control group		NPGN vs. control group	
	Mean ± SD	Median (range)	Mean ± SD	Median (range)	Mean ± SD	Median (range)	*t*/*Z*	*p* value	*t*/*Z*	*p* value	*t*/*Z*	*p* value
Leukocytosis (×10^3^ cells/μL)	7.3 ± 2.7	6.9 (3.6–11.1)	6.6 ± 1.5	6.4 (4.9–9.8)	6.8 ± 0.4	6.7 (6.3–7.6)	0.7	0.48	0.8	0.44	−0.63	0.53
RBC (×10^6^ cells/μL)	4.3 ± 0.6	4.3 (3.3–5.2)	4.3 ± 0.5	4.5 (3.7–5.1)	5.2 ± 0.4	5.1 (4.5–5.8)	−0.09	0.93	−4.7	<0.0001	−4.9	<0.0001
Haemoglobin (g/dL)	12.4 ± 1.8	12.5 (9.3–15.1)	13.5 ± 1.5	13.5 (11.0–15.7)	14.3 ± 1.2	14.4 (12.5–16.9)	−1.4	0.19	−3.3	0.0024	−1.7	0.11
Platelets (×10^3^ cells/μL)	248.3 ± 82.1	214.5 (177.0–410.0)	217.7 ± 47.9	209.0 (120.0–305.0)	279.0 ± 57.0	281.5 (186.0–403.0)	1.0	0.32	−1.20	0.24	−2.9	0.0069

*PGN* proliferative glomerulonephritis, *NPGN* non-proliferative glomerulonephritis, *RBC* red blood cells, *t/Z* Student’s *t*-distribution/*Z* value

**Table 2:** Comparison of renal function parameters in PGN patients, NPGN patients and control group

Parameters	PGN		NPGN		Control group		Group of patients					
							PGN vs. NPGN		PGN vs. control group		NPGN vs. control group	
	Mean ± SD	Median (range)	Mean ± SD	Median (range)	Mean ± SD	Median (range)	*t*/*Z*	*p* value	*t*/*Z*	*p* value	*t*/*Z*	*p* value
Blood urea nitrogen (mg/dL)	22.2 ± 12.6	19.6 (11.6–54.0)	22.8 ± 14.3	17.8 (9.1–50.6)	14.7 ± 3.2	14.95 (8.4–19.6)	−0.1	0.92	2.6	0.016	2.5	0.020
Serum creatinine (mg/dL)	1.09 ± 0.49	0.91 (0.68–2.3)	1.11 ± 0.48	0.96 (0.47–2.27)	0.92 ± 0.12	0.93 (0.7–1.13)	−0.5	0.60	0.0	1.0	1.2	0.22
Serum uric acid (mg/dL)	6.4 ± 1.3	6.8 (4.0–8.0)	6.2 ± 1.1	5.8 (5.3–8.6)	6.2 ± 1.4	6.95 (3.7–7.9)	0.7	0.47	0.4	0.72	0.09	0.93
Total quantity of protein in 24-h urine collection test	5.1 ± 3.4	3.6 (1.6–12.1)	5.06 ± 1.8	4.8 (1.2–7.2)	0.0 ± 0.0	0.0 (0.0)	−0.7	0.47	4.4	<0.0001	4.4	<0.0001

**Table 3:** Levels of selected proteins and complement components in PGN patients, NPGN patients and healthy individuals

Parameters	PGN		NPGN		Control group		Group of patients					
							PGN vs. NPGN		PGN vs. control group		NPGN vs. control group	
	Mean ± SD	Median (range)	Mean ± SD	Median (range)	Mean ± SD	Median (range)	*t*/*Z*	*p* value	*t*/*Z*	*p* value	*t*/*Z*	*p* value
Serum IgG concentration (g/L)	5.0 ± 1.7	5.3 (2.2–6.9)	4.9 ± 2.2	3.9 (3.2–9.0)	12.7 ± 1.4	12.8 (10.1–15.5)	0.06	0.96	−13.3	<0.0001	−11.9	<0.0001
Serum IgM concentration (g/L)	0.99 ± 0.46	0.97(0.4–1.72)	1.15 ± 0.59	1.04 (0.5–2.4)	1.7 ± 0.3	1.6 (1.2–2.2)	−0.7	0.51	−4.7	<0.0001	−3.2	0.0038
Serum IgA concentration (g/L)	2.7 ± 1.2	3.0 (0.93–4.5)	2.5 ± 1.1	2.3 (1.3–4.7)	2.4 ± 0.84	2.6 (0.9–3.9)	0.4	0.73	0.7	0.46	0.27	0.78
Serum total protein (g/L)	4.8 ± 0.9	5.1 (3.2–6.0)	4.6 ± 0.7	4.5 (3.5–5.6)	7.4 ± 0.6	7.4 (6.4–8.2)	0.6	0.58	−9.5	<0.0001	−11.5	<0.0001
Serum albumin (g/L)	2.7 ± 0.8	2.9 (1.4–3.9)	2.5 ± 0.6	2.5 (1.8–3.8)	4.2 ± 0.36	4.2 (3.5–4.75)	0.4	0.67	−7.2	<0.0001	−9.2	<0.0001
Serum alpha 1 globulins (g/L)	0.14 ± 0.07	0.1 (0.1–0.3)	0.12 ± 0.04	0.1 (0.1–0.2)	0.16 ± 0.05	0.20 (0.1–0.2)	0.34	0.73	−1.1	0.28	−1.5	0.077
Serum alpha 2 globulins (g/L)	0.74 ± 0.15	0.7 (0.59–1.1)	0.78 ± 0.14	0.8 (0.5–1.0)	0.96 ± 0.16	0.99 (0.65–1.18)	−0.6	0.58	−3.6	0.0013	−3.0	0.0053
Serum beta globulins (g/L)	0.45 ± 0.15	0.45 (0.2–0.6)	0.48 ± 0.09	0.48 (0.4–0.71)	0.60 ± 0.1	0.61 (0.42–0.75)	−0.6	0.58	−3.3	0.0027	−3.1	0.0046
Serum gamma globulins (g/L)	0.34 ± 0.12	0.35 (0.2–0.5)	0.30 ± 0.12	0.3 (0.2–0.6)	0.36 ± 0.09	0.35 (0.24–0.50)	0.8	0.45	−0.6	0.57	−1.6	0.11
Serum complement component C3 (g/L)	1.20 ± 0.17	1.21 (1.0–1.5)	1.19 ± 0.44	1.2 (0.4–2.0)	1.28 ± 0.22	1.24 (0.95–1.78)	0.05	0.96	−1.1	0.29	−0.77	0.45
Serum complement component C4 (g/L)	0.29 ± 0.07	0.27 (0.23–0.46)	0.30 ± 0.10	0.3 (0.11–0.45)	0.28 ± 0.08	0.29 (0.15–0.39)	−0.3	0.76	0.30	0.77	0.63	0.54

### Frequencies of PD-1-Positive and PD-L1-Positive T and B Lymphocytes in PGN Patients, NPGN Patients and Control Group

**Fig. 2:**
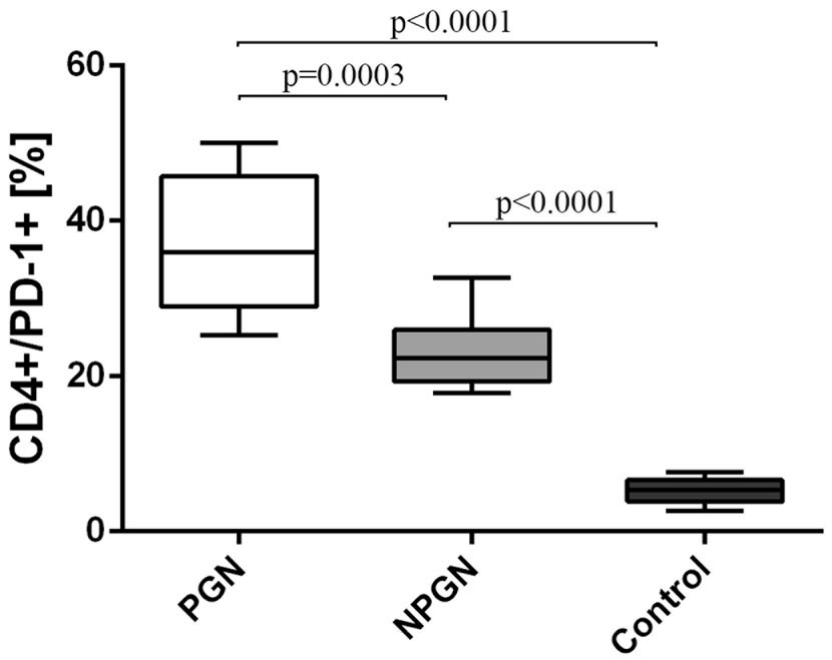
The frequencies of CD4^+^/PD-1^+^ cells in PGN patients, NPGN patients and healthy volunteers

Using the flow cytometric analysis, we assessed the differences in the frequencies of PD-1-positive and PD-L1-positive T and B lymphocytes in study groups and healthy individuals. The frequencies of CD4^+^/PD-1^+^ T cells were higher among patients suffering from PGN (36.8 ± 8.6%) [median 36.0% (25.2–50.1%)] and NPGN (23.2 ± 4.5%) [median 22.3% (17.8–32.6%)] than in the control group (5.4 ± 1.5%) [median 5.3% (2.7–7.7%)] (Fig. 2).

**Fig. 3:**
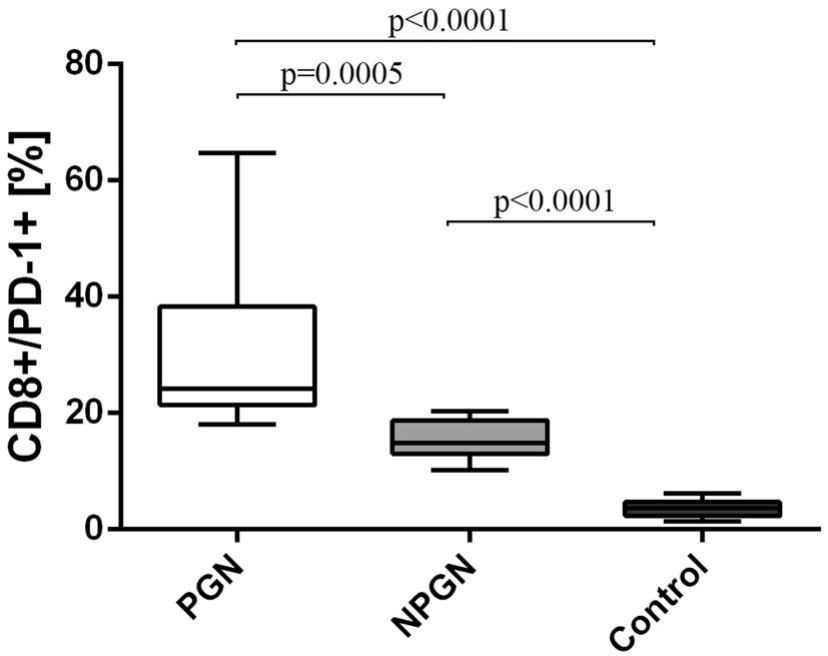
The frequencies of CD8^+^/PD-1^+^ cells in PGN patients, NPGN patients and healthy volunteers

Similarly, the frequencies of CD8^+^/PD-1^+^ T cells in the PGN group (30.4 ± 14.6%) [median 24.2% (18.0–64.8%)] exceeded both the NPGN group (15.4 ± 3.3%) [median 14.8% (10.2–20.3%)] and the control group (3.6 ± 1.5%) [median: 3.7% (1.4–6.2%)] (Fig. 3).

**Fig. 4:**
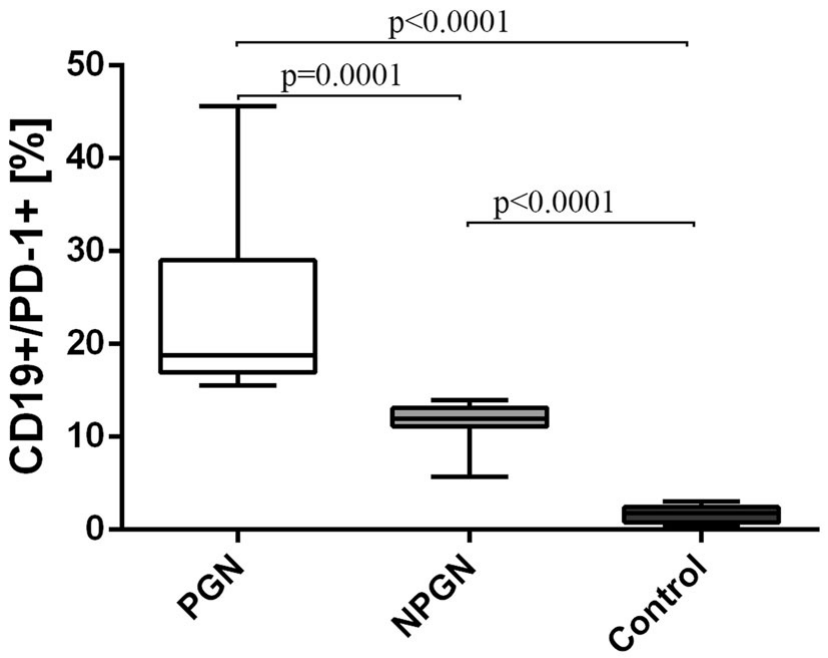
The frequencies of CD19^+^/PD-1^+^ cells in PGN patients, NPGN patients and healthy volunteers

The frequencies of CD19^+^/PD-1^+^ B cells were also higher in both patients suffering from PGN (23.6 ± 9.5%) [median 18.8% (15.6–45.6%)] and NPGN (11.6 ± 2.4%) [median 12.0% (5.7–14.0%)] than in healthy subjects (1.67 ± 0.84%) [median 1.8% (0.4–3.0%)] (Fig. 4).

The frequencies of all the above-mentioned cells were also significantly higher in PGN group than in the NPGN group (Figs. 2, 3, 4, respectively).

**Fig. 5:**
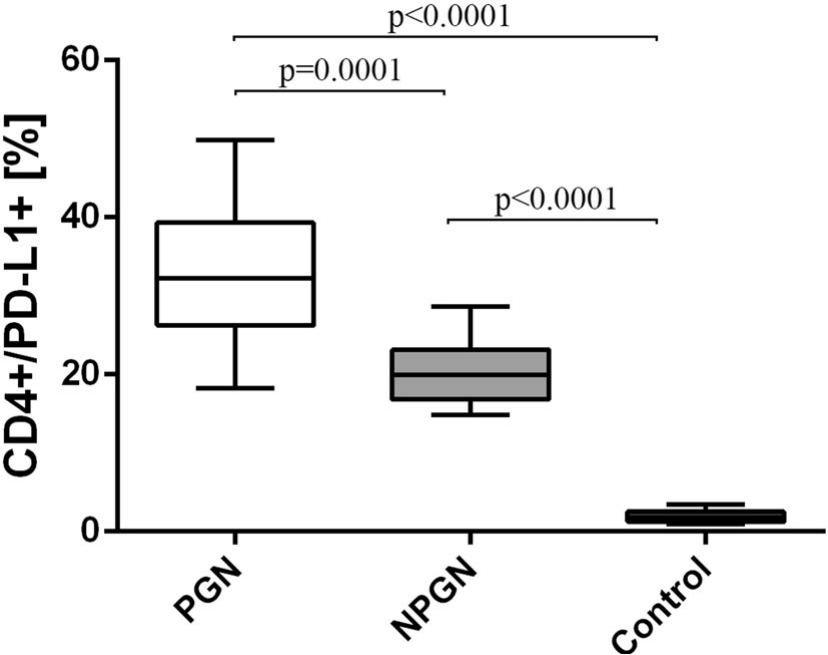
The frequencies of CD4^+^/PD-L1^+^ cells in PGN patients, NPGN patients and healthy volunteers

We observed the same pattern, when looking at the frequencies of the PD-L1-positive cells. The frequencies of CD4^+^/PD-L1^+^ T cells in patients with PGN (32.9 ± 9.3%) [median 32.2% (18.2–49.8%)] and with NPGN (20.4 ± 4.3%) [median 20.0% (14.8–28.7%)] were statistically significantly higher than in the control group (1.86 ± 0.70%) [median 1.7% (1.0–3.5%)] (Fig. 5). In our study, the PGN patients presented a higher occurrence of those cells than the NPGN patients (Fig. 5).

**Fig. 6:**
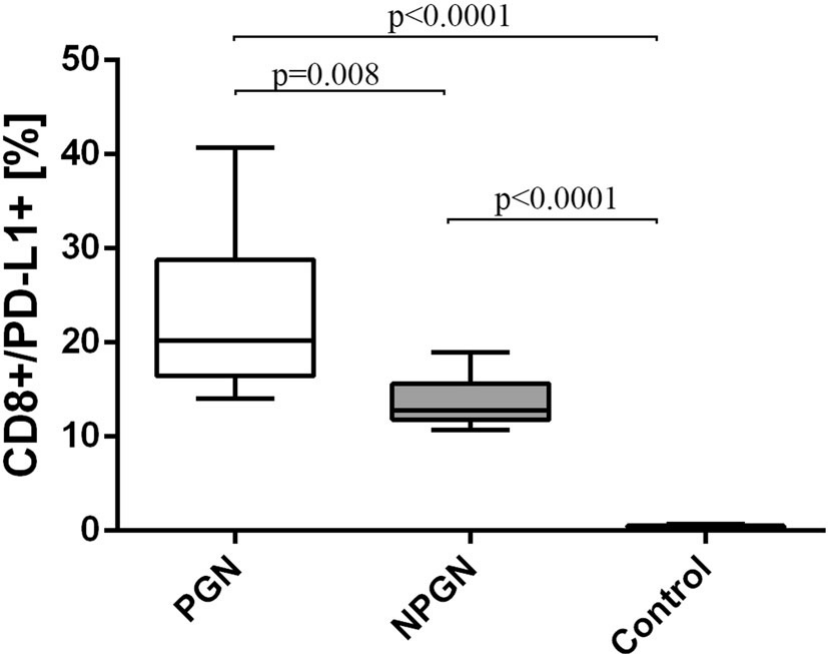
The frequencies of CD8^+^/PD-L1^+^ cells in PGN patients, NPGN patients and healthy volunteers

Likewise, the frequencies of CD8^+^/PD-L1^+^ T cells were higher in patients from PGN group (23.1 ± 9.6%) [median 20.2% (14.0–40.7%)] and NPGN group (13.7 ± 2.6%) [median 12.8% (10.7–19.0%)] than in healthy individuals (0.45 ± 0.11%) [median 0.43% (0.31–0.67%)] (Fig. 6). Patients from PGN group showed a higher incidence of those cells than patients from NPGN group (Fig. 6).

**Fig. 7:**
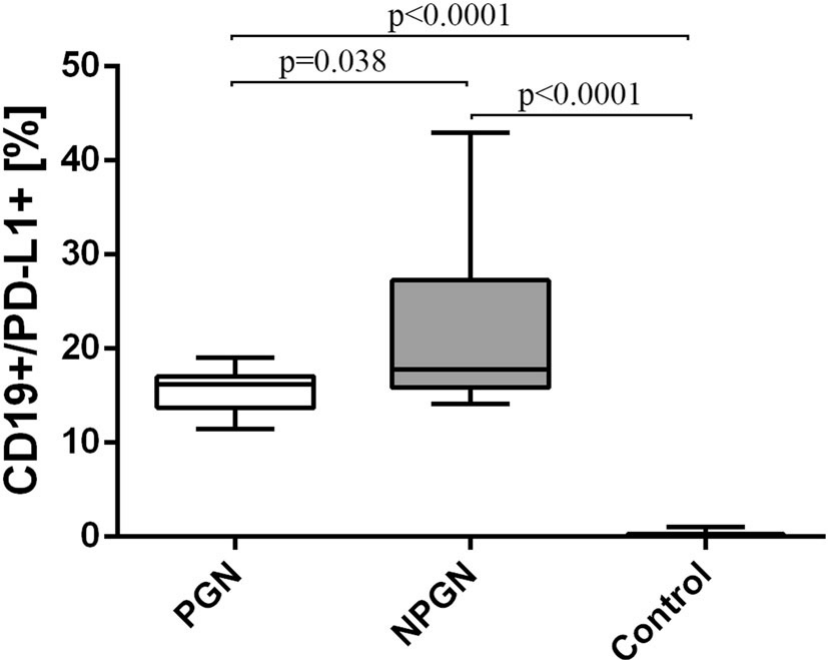
The frequencies of CD19^+^/PD-L1^+^ cells in PGN patients, NPGN patients and healthy volunteers

Finally, the frequencies of CD19^+^/PD-L1^+^ B cells were higher in patients with PGN (22.3 ± 8.8%) [median 17.8% (14.1–43.0%)] than in patients with NPGN (15.6 ± 2.3%) [median 16.2% (11.5–19.1%)] (Fig. 7). Both examined groups showed higher frequencies of those cells than the control group (Fig. 7).

**Fig. 8:**
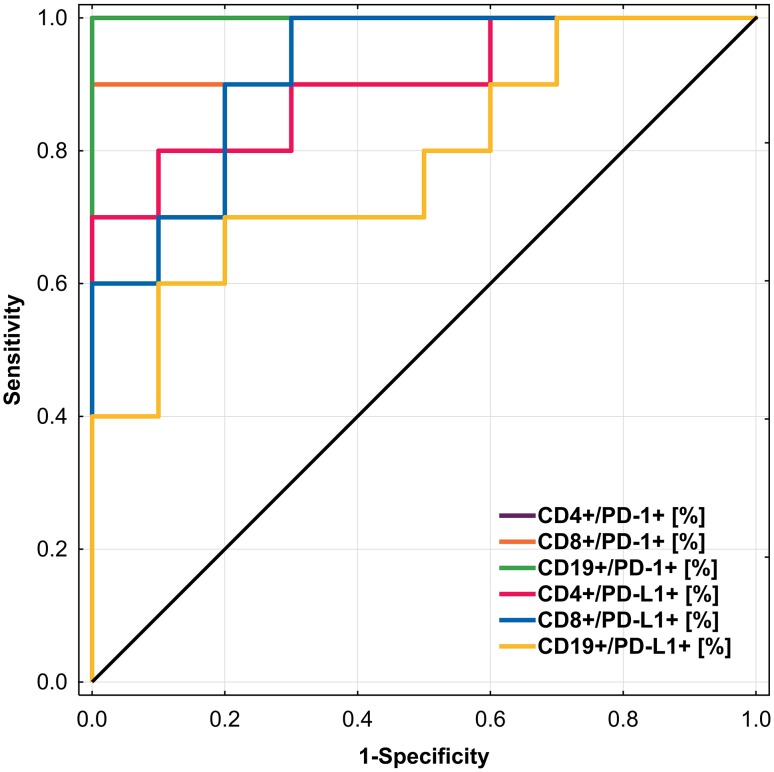
ROC curve comparing the sensitivity and specificity of immunological parameters in patients with PGN and NPGN

Figure 8 and Table 4 shows the ROC analysis of the six immunological parameters. As the AUC shows, the percentage of CD19^+^/PD-1^+^ cells was the most sensitive and specific parameter to determine patients with NPGN (AUC = 1.0).

**Table 4:** ROC analysis to determine diagnostic accuracy in differentiation of PGN and NPGN patients

Parameter	Prognostic value	AUC	95% CI
CD4^+^/PD-1^+^ (%)	29.48	0.93	0.82–1.0
CD8^+^/PD-1^+^ (%)	20.41	0.97	0.90–1.0
CD19^+^/PD-1^+^ (%)	15.56	1.0	1.0
CD4^+^/PD-L1^+^ (%)	27.73	0.9	0.76–1.0
CD8^+^/PD-L1^+^ (%)	15.51	0.92	0.80–1.0
CD19^+^/PD-L1^+^ (%)	17.83	0.78	0.57–0.99

*95% CI* 95% confidence interval

### Correlations

#### Correlations in Patients with PGN

In the group of patients with PGN, we have found a positive correlation between the frequencies of CD4^+^/PD-1^+^ and CD4^+^/PD-L1^+^ T cells (0.95; *p* < 0.0001). We have also found a positive correlation between the frequencies of CD4^+^/PD-1^+^ and CD8^+^/PD-L1^+^ T cells (0.70; *p* = 0.024). Moreover, a positive correlation was stated between the frequencies of CD8^+^/PD-1^+^ T cells and the frequencies of CD8^+^/PD-L1^+^ T cells in those patients (0.91; *p* < 0.0001). Finally, the frequencies of CD19^+^/PD-1^+^ B cells positively correlated with the frequencies of CD19^+^/PD-L1^+^ (0.98; *p* < 0.0001).

#### Correlations in Patients with NPGN

Next, the group of patients with NPGN was checked, and a positive correlation between frequencies of CD4^+^/PD-1^+^ T cells and CD4^+^/PD-L1^+^ T cells (0.87; *p* < 0.0001) was found. A positive correlation was also stated between the frequencies of CD8^+^/PD-1^+^ and CD8^+^PD-L1^+^ T cells (0.82; *p* = 0.003). Moreover, we have found a positive correlation between the frequencies of CD19^+^/PD-1^+^ B cells and the frequencies of CD19^+^/PD-L1^+^ B cells in those patients (0.80; *p* = 0.005).

#### Correlations in the Control Group

No statistically significant correlations were found in the healthy subjects.

### Correlations of PD-1-Positive and PD-L1-Positive T and B Lymphocytes with Selected Laboratory Parameters

#### Correlations in Patients with PGN

In patients with PGN, we have found a positive correlation between the platelet (PLT) count and the frequencies of CD19^+^/PD-1^+^ B cells (0.89; *p* = 0.001), and also with the frequencies of CD19^+^/PD-L1^+^ B cells (0.88; *p* = 0.001). Moreover, a statistically significant positive correlation was found between the level of serum beta globulins and the frequencies of CD4^+^/PD-1^+^ T cells (0.73; *p* = 0.016), CD19^+^/PD-1^+^ B cells (0.68; *p* = 0.03), and CD19^+^/PD-L1^+^ B cells (0.67; *p* = 0.033).

#### Correlations in Patients with NPGN

In patients with NPGN, a statistically significant negative correlation was stated between the blood urea nitrogen (BUN) and the frequencies of CD8^+^/PD-L1^+^ T cells (–0.66; *p* = 0.037). Moreover, the concentration of serum IgM showed a significant positive correlation with the frequencies of CD4^+^/PD-1^+^ T cells (0.75; *p* = 0.012) and the frequencies of CD4^+^/PD-L1^+^ T cells (0.70; *p* = 0.023).

#### Correlations in the Control Group

No statistically significant correlations were found in the healthy subjects.

## Discussion

PD-1 is a member of the CD28/cytotoxic T-lymphocyte-associated protein-4 superfamily, which plays an important role in the regulation of activated T cells (Odorizzi et al. 2015). However, it is not clear how PD-1 is expressed in a normal and diseased kidney, nor if it has a role in progression of chronic renal disease. The results of the studies are inconclusive. Autoreactive lymphocytes are suppressed in healthy individuals by the so-called peripheral tolerance. Accumulating evidence indicates that co-receptor signalling plays a pivotal role in the regulation of autoreactive lymphocytes. The positive regulatory co-receptors CD28 and inducible co-stimulator (ICOS) transduce stimulatory co-signals, whereas the negative regulatory co-stimulators CTLA-4 and PD-1 are critical for the regulation of peripheral tolerance and autoimmunity (Nishimura et al. 1999; Shi et al. 2016).

Our study revealed that the frequencies of PD-1-positive and PD-L1-positive T and B lymphocytes were higher among patients suffering from PGN than in patients with diagnosed NPGN, and higher than in the control group. Moreover, we found that the frequencies of PD-1-positive T CD4^+^ lymphocytes strongly correlated with PD-L1-positive T CD4^+^ lymphocytes, PD-1-positive T CD8^+^ lymphocytes strongly correlated with PD-L1-positive T CD8^+^ lymphocytes, and PD-1-positive B CD19^+^ lymphocytes strongly correlated with PD-L1-positive B CD19^+^ lymphocytes. These findings were particularly significant in the group of PGN patients and absent in the control group.

In an animal model Nishimura et al. (1999) observed that introduction of a PD-1 null mutation into the 2C-TCR (anti-H-2Ld) transgenic mice of the H-2(b/d) background resulted in the chronic and systemic graft-versus-host-like disease. Furthermore, CD8^+^ 2C-TCR^+^ PD-1^−/−^ T cells exhibited markedly augmented proliferation in vitro in response to H-2d allogeneic cells. Collectively, it was suggested that PD-1 is involved in the maintenance of peripheral self-tolerance by serving as a negative regulator of immune responses (Nishimura et al. 1999). PD-1 deficient mice developed lupus-like glomerulonephritis and arthritis on a C57Bl/6 background and autoimmune-dilated cardiomyopathy on a BALB/c background (Okazaki et al. 2002). Ding et al. (2006) developed a recombinant adenovirus containing the full-length mouse PD-L1 gene (Ad.PD-L1) to engage the immunoinhibitory receptor PD-1 on activated lymphocytes to prevent lupus nephritis in BXSB mice. This strategy was further reinforced by concomitant injection of anti-ICOSL(B7h) mAb to block ICOS-mediated co-stimulation. The combined therapy dramatically delayed the onset of proteinuria, effectively inhibited IgG autoantibody production, and significantly reduced hypercellularity and deposition of IgG in glomeruli, resulting in almost complete amelioration of lupus nephritis in these animals. Those findings indicated the therapeutic potential of simultaneous stimulation of PD-1-mediated pathway and blockade of ICOS-B7h co-stimulation in the prevention of lupus nephritis (Ding et al. 2006). The other study revealed that blockade of PD-1 worsened progressive renal histopathological and functional injury in murine adriamycin nephropathy, which suggested a possible protective role for PD-1 in chronic renal disease, and its potential as a treatment to slow the disease progression (Qin et al. 2006). To investigate the role of the PD-1/PDL-1 co-inhibitory pathway in development of experimental autoimmune glomerulonephritis, the in vivo effects of a stimulating PDL-1/Fc fusion protein were examined after the onset of the disease in rats (Reynolds et al. 2012). Authors demonstrated that stimulation of PD-1 led to a significant reduction in albuminuria, serum urea, serum creatinine, crescent formation and tubular damage compared with controls. There was also a reduction in numbers of glomerular macrophages, CD4^+^ T cells, CD8^+^ T cells and PD1^+^ cells compared with controls (Reynolds et al. 2012). Blocking PD-1 did not increase serum antigen-specific antibodies or increase glomerular immunoglobulin G deposition, the hallmark of injury in proliferative immune complex glomerulonephritis of BALB/c mice, caused by injection of horse spleen apoferritin (Ooi et al. 2015). Furthermore, C3 deposition was unaffected and glomerular macrophages were reduced after anti-PD-1 antibodies (Ooi et al. 2015). However, anti-PD-1 administration increased splenocyte proliferation and cytokine production including interferon (IFN)-γ, interleukin (IL)-4, and IL-17. Neutralizing either PD-L1 or PD-L2 alone did not result in major alterations in renal injury (Ooi et al. 2015). It was also observed that the frequency of PD-1^+^/CXCR5^+^ follicular helper T (TFH) cells was significantly increased in BXD2 mice, which spontaneously develop autoantibodies and subsequent glomerulonephritis, offering a useful animal model to study autoimmune lupus, compared with wild-type mice (Kim et al. 2015).

Data in humans are focused mainly on secondary glomerulonephritides. Research performed by Zhang et al. (2006) revealed that PD-1 ligand is specifically expressed on renal tubular epithelial cells in diseased human kidney samples, including lupus nephritis, tubulointerstitial nephritis and renal cell carcinoma. PD-1 ligand was described as a strong inhibitor of CD4^+^ T-cell activation, as assessed by increased cytokine (IFN-γ and IL-2) production and enhanced levels of T-cell activation marker CD69 (Zhang et al. 2006). The expansion of circulating TFH cells (CD4^+^/CXCR5^+^/ICOS^+high^/PD-1^+high^) has been described in patients with lupus glomerulonephritis and cytopenias (Gómez-Martín et al. 2011). Despite the fact that research concerned secondary nephropathy, Liu et al. (2014) obtained similar results to us. Significantly higher frequency of CD4^+^/CXCR5^+^, CD4^+^/CXCR5^+^/ICOS^+^ and CD4^+^/CXCR5^+^/PD-1^+^ TFH cells, and higher serum levels of IL-17A, IFN-γ, IL-2, IL-10, IL-4 and IL-21 were detected in hepatitis B virus-associated membranous nephropathy patients compared to the healthy controls (Liu et al. 2014). Notably, the percentage of CD4^+^/CXCR5^+^/PD-1^+^ TFH cells was positively correlated with serum IL-21 level and 24-h urinary protein concentration (Liu et al. 2014). Treatment with prednisone or/and immunosuppressive drugs significantly reduced the frequency of CD4^+^/CXCR5^+^ CD4^+^CXCR5^+^/ICOS^+^ and CD4^+^/CXCR5^+^/PD-1^+^ TFH cells (Liu et al. 2014). Recent results obtained by Shi et al. (2016) in the group of patients with idiopathic membranous nephropathy (IMN) seems to be also in line with our outcomes as the frequencies of total, ICOS^+^, and PD-1^+^ TFH cells were increased in IMN patients. What is more, the ratio of ICOS^+^/PD-1^+^ TFH cells positively correlated with IMN progression (Shi et al. 2016). In the case of IgA nephropathy results were similar (Zhang et al. 2014). Moreover, treatment with prednisone significantly reduced the frequency of CD4^+^/CXCR5^+^ and CD4^+^/CXCR5^+^/PD-1^+^ TFH cells and the levels of serum IL-21, but increased IL-4 and IL-10 in those patients (Zhang et al. 2014).

Our study sheds new light on the role of PD-1/PD-L1 pathway in the pathogenesis of different types of GN as the percentages of PD-1^+^ cells, especially CD19^+^/PD-1^+^ cells, were sensitive and specific parameter to determine patients with PGN and NPGN. Analyzed molecules should be, therefore, considered as a valuable diagnostic tool.

Our team also found a statistically significant positive correlation between the level of serum beta globulins and the frequencies of CD4^+^/PD-1^+^ T cells, CD19^+^/PD-1^+^ B cells, and CD19^+^/PD-L1^+^ B cells in patients with PGN. As beta globulin fraction is composed mainly of transferrin and C3 complement component, it seems likely that widely postulated role of C3 in the development of PGN is a consequence of inappropriate immune system activation due to the over-expression of PD-1 and PD-L1 antigens (Ito et al. 2016; Wijnen and van Dieijen-Visser 1996). IgA nephropathy is often accompanied with C3 deposits in glomeruli and some authors describe C3 serum level as a predictor of PGN (Liu et al. 2016; Tomino 2014). It is worth to notice that the relationships are only observed between B cells and T CD4^+^ cells (but not T CD8^+^ cells), and serum beta globulins level. In the pathogenesis of PGN, therefore, cytotoxic reactions are probably less impaired than antigen recognition and antibody secretion.

In patients with PGN, we have found a positive correlation between the PLT count and the frequencies of CD19^+^/PD-1^+^ B cells, and also with the frequencies of CD19^+^/PD-L1^+^ B cells. It was proven that CKD patients have significant PLT activation and endothelial dysfunction, which was involved in CKD’s occurrence and development (Lu et al. 2015). Pro-inflammatory factors released from PLT are responsible for glomerular injury (van Roeyen et al. 2011). PD-1/PD-L1 levels may have a role in PLT activation as without the inhibitory regulation of PD-1, sustained activation of T cells may cause inflammatory responses. Similar observations were recently described in the case of immune thrombocytopenia (Birtas Atesoglu et al. 2016).

Analysis of correlations between laboratory parameters and PD-1/PD-L1 expression showed that in patients with NPGN the concentration of serum IgM was significantly positively correlated with the frequencies of CD4^+^/PD-1^+^ T cells and the frequencies of CD4^+^/PD-L1^+^ T cells. Moreover, a statistically significant negative correlation was stated between the BUN and the frequencies of CD8^+^/PD-L1^+^ T cells. Most serum IgM is produced apparently spontaneously by a distinct subset of B cells requiring no exogenous antigenic or microbial stimuli (Nguyen and Baumgarth 2016). Natural IgM is an evolutionarily conserved molecule and reacts with a variety of epitopes expressed on both self- and non-self antigens (Nguyen and Baumgarth 2016). It has long been understood that secreted IgM contributes to the removal of altered self-antigens, such as apoptotic and dying cells (Nguyen and Baumgarth 2016). IgM is also critical for B-cell central tolerance induction (Nguyen and Baumgarth 2016). PD-1/PD-L1 pathway-induced inhibition of the immune response may be responsible for inappropriate function of IgM, i.e. loss of B-cell central tolerance and accumulation of altered self-antigens, and glomerular injury in NPGN.

Therefore, our and other authors’ researches suggest that PD-1-blockade might be an important treatment option for patients with GN. On the other hand, two different forms of immune checkpoint inhibitor-induced renal damage have been identified, including acute (granulomatous) tubulointerstitial nephritis and immune complex glomerulonephritis as a result of treatment of several malignancies with ipilimumab and tremelimumab (anti-CTLA-4-blocking antibodies), and pembrolizumab and nivolumab (antibodies targeting PD-1 receptors) (Izzedine et al. 2017).

In conclusion, our results suggest that deregulation of PD-1/PD-L1 axis may contribute the PGN and NPGN pathogenesis. Alteration of PD-1/PD-L1 pathway may be involved in poorer prognosis, as patients with PGN are characterized by higher frequencies of PD-1-positive and PD-L1-positive T and B lymphocytes than patients with NPGN. High percentages of lymphocytes with PD-1 and PD-L1 expression may contribute to the continuous T-cell activation and development of glomerular inflammation and injury. Further studies, currently under investigation by our team, are needed to explain if similar pathologies are found in the glomeruli of PGN and NPGN patients, and if PD-1/PD-L1 blockade is able to induce long-term remission of PGN and NPGN.

In this work, we presented findings in a relatively small study group. Further research should include assessment of PD-1 and PD-L1 expression on more cell populations, including antigen-presenting cells in PB and on glomeruli, as well as glomerular cell infiltrates. The study should be continued and the expression of PD-1 and PD-L1 has to be evaluated during the treatment of GN, and after obtaining remission.

## Abbreviations

CKD: Chronic kidney disease
ESRD: End-stage renal disease
GN: Glomerulonephritides
NPGN: Primary non-proliferative glomerulonephritides
PB: Peripheral blood
PD-1: Programmed cell death protein 1
PD-L1: Programmed cell death protein ligand 1
PGN: Primary proliferative glomerulonephritides
TCR: T-cell receptor
Th: T helper cell
ROC: Receiver operating characteristic curve
AUCs: Areas under the ROC curves
ICOS: Inducible co-stimulator
IFN: Interferon
IL: Interleukin
TFH: Follicular helper T cells
